# Do medical students and young physicians assess reliably their self-efficacy regarding communication skills? A prospective study from end of medical school until end of internship

**DOI:** 10.1186/s12909-017-0943-y

**Published:** 2017-06-30

**Authors:** Tore Gude, Arnstein Finset, Tor Anvik, Anders Bærheim, Ole Bernt Fasmer, Hilde Grimstad, Per Vaglum

**Affiliations:** 1 Department of Behavioural Sciences in Medicine, Institute of Basic Medical Sciences, Faculty of Medicine, University of Oslo, POB 1111 – Blindern, Norway, N-0317 Oslo, Norway; 2 Department of Community Medicine, Faculty of Health Sciences, University of Tromsø, Tromsø, Norway; 3 Department of Global Public Health and Primary Care, Faculty of Medicine and Dentistry, University of Bergen, Bergen, Norway; 4 Department of Clinical Medicine, Section for Psychiatry, Faculty of Medicine and Dentistry, University of Bergen, Bergen, Norway; 5 Department of Public Health and General Practice, Faculty of Medicine, Norwegian University of Science and Technology, Trondheim, Norway

**Keywords:** Communication skills, Self-efficacy, Medical consultations, Young physicians

## Abstract

**Background:**

This prospective study from end of medical school through internship investigates the course and possible change of self- reported self-efficacy in communication skills compared with observers’ ratings of such skills in consultations with simulated patients.

**Methods:**

Sixty-two medical students (43 females) from four Norwegian universities performed a videotaped consultation with a simulated patient immediately before medical school graduation (T1) and after internship (internal medicine, surgery and family medicine, half a year each - T2). Before each consultation, the participants assessed their general self-efficacy in communication skills. Trained observers scored the videos and applied a well-validated instrument to rate the communication behaviour. Results from the two assessment methods were correlated at both time points and possible differences from T1 to T2 were explored.

**Results:**

A close to zero correlation between self-efficacy and observed communication skills were found at T1. At T2, participants’ self-efficacy scores were inversely correlated with levels of observed skills, demonstrating a lack of concordance between young physicians’ own assessment of self-efficacy and observers’ assessment. When dividing the sample in three groups based on the observers’ scores (low <1/3-, medium 1/3 to 2/3-, high competence >2/3), the group of male physicians showed higher levels of self-efficacy than females in all the three performance groups at T1. At T2, those having a high performance score yielded a low self-efficacy, regardless of gender.

**Conclusions:**

The lack of positive correlations between self-efficacy assessment and expert ratings points to limitations in the applicability of self-assessment measures of communication skills. Due to gender differences, groups of female and male physicians should be investigated separately. Those obtaining high-performance ratings from observers, through the period of internship, may become more conscious of how demanding clinical communication with patients may be. This insight may represent a potential for growth, but could in some physicians represent too much of a self-critical attitude. Active supervision of young physicians throughout internship is important in order to help physicians to be more aware of their strengths and weaknesses, in order to gain increased mastery in the art of doctoring.

## Background

In research on physician-patient relations, communication skills are most often assessed by methods of direct observation of the dialogue between the two parties [1]. Consultations are observed in real time or recorded on audio- or videotape, and communication behaviour is coded according to an interaction analysis system, scored according to a rating scale, or described and analysed with the application of qualitative methods. All these methods are, to varying extents, elaborate and time-consuming. Monitoring of medical students’ and physicians’ communication skills are an important part of training in both pre- and post-graduation curricula. Therefore, less resource-consuming ways of performing such evaluation should be considered. One possibility could simply be to ask physicians or students to assess their self-competence concerning communication skills, or to evaluate the level of competence reached in a training programme or after a specific performance in various courses of communication skills training. If methods based on self-assessment were valid and stable indicators of the quality of communication skills, they would have the advantages of being quicker and easier to perform.

However, research has demonstrated varying degrees of discrepancy between self-assessment and observers’ ratings of corresponding skills. Ward et al. identified 62 studies, covering the assessment of a broad range of skills, including clinical communication. They found that the concordance of self-assessment towards expert ratings tended to be poor, and concluded that prior research in this area must be re-examined in light of common weaknesses in study design and analysis methods [2]. The main bulk of studies referred by Ward et al. used correlation analyses only in their methodology. In our study, we have in addition performed One-way analyses of variance to detect gender differences.

Davis et al. reviewed studies of concordance between physicians’ self-assessment and observed measures of competence in different areas of performance. Of 20 studies comparing self- and external-assessment, 13 demonstrated little, no or an inverse relationship, while seven demonstrated positive correlations [3]. The authors concluded that the lowest degree of accuracy in self-assessment tended to occur among the least-skilled physicians who also reported most confidence in themselves.

When it comes to studies explicitly assessing clinical communication skills, different findings have been reported. Gruppen et al. found a correlation of .22 between fourth year medical students’ self-ratings and standardized patients’ assessments of seven basic clinical skills [4]. Gordon found correlations ranging from .31 to .64 between students’ or physicians’ self-reported skills and scores from expert observers in four studies using video reviews [5].

Most of the studies referred to above measured medical students’ or physicians’ self-assessments after a specific task (consultation, role-play or the Objective Structured Clinical Examination) that had been committed and thus represented a self-assessment of their performance on that specific task, but not as a measure of competence in communication skills in general.

This approach has been used in studies with focus on students or young doctors assessing their confidence in performing skills independent of a specific task. In the literature, this confidence in one’s own performance is referred to as “self-efficacy”, a concept that was introduced by Bandura [6] and adopted by other researchers [7].

In this field, findings are also inconsistent. Jenkins and Fallowfield found that changes in physicians’ communication behaviour after training concurred with changes in their self-assessment [8]. Gulbrandsen et al. [9] found no significant association between self-efficacy scores and competence based on observers’ ratings of consultations with real patients using the Four Habits Coding Scheme [10] before training in “Four Habits” [11]. However, they did find a significant positive correlation, although relatively weak, at the three-year follow-up [9]. Therefore, we could expect the confidence level of a graduating student to change when becoming more experienced during their internship, a period in their professional life with massive exposure to clinical practice. If this assumption does not hold true, it is necessary to perform a closer investigation of the issue in order to possibly explain the discrepancy between self-assessment and observers’ ratings.

As to gender differences, females generally tend to assess their own skills less favourably than males do [12, 13], and similar findings have been reported in medicine. For instance, Bakken et al. found that female physicians consistently rated their abilities to apply skills in clinical research at a lower level than males did [14], whereas other studies report that male and female physicians display different levels of confidence in different areas of performance [15, 16].

With this background, we have conducted a study that aims to explore the possibility of using students’ and young physicians’ self-assessed self-efficacy as an evaluation measure of competence. In a combined cross-sectional and a prospective study design, we wanted to investigate the relationship between their opinion about their general competence in communication skills and observers’ ratings of these skills based on videotaped consultations with simulated patients. Should a negative or no relationship be found, we aimed at exploring more in detail any differences from end of medical school (T1) until end of internship (T2). We also wanted to explore whether female and male physicians would show different patterns of relationship between the two assessment forms.

Therefore, our aims were the following:

1. What are the levels of and the relationships between self-reported self-efficacy in communication skills and independent observers’ ratings of such skills in videotaped consultations in the same cohort at the end of medical school (T1) and after internship 2 years later (T2)?
2. Will this relationship be different at the two assessments?
3. Will gender differences occur?

## Methods

### Subjects

In order to evaluate the level of communication skills in medical students after training throughout the curriculum, those comprising a 1 year-cohort (*N* = 320, mean age 27.3 ± 2.7, range 21–41) in all four medical schools in Norway in 2004 were invited to perform a consultation with a simulated patient a couple of months before graduation. One-hundred and eleven (34.7%) students agreed to take part in the study (mean age 28.3 ± 3.0, range 23–45, 70% women). Among them, 93% had worked as an assistant physician during the last part of medical school, mainly in hospitals. Not all of the students continued directly from medical school into the obligatory 18 months of internship (half a year of internal medicine, half a year in surgery, and half a year in a GP setting) due to reasons like child birth, lack of jobs within the organised internship, or need for a break. Those among the 111 attendees at T1 who proceeded directly into internship (*N* = 78), were invited to perform another consultation at the end of this period being approximately 2 years after the first performance (2006- T2). Of them, 75 young physicians agreed to participate, but logistic and mail delivery problems reduced the total number to 62 physicians who participated in the follow-up consultation, a reduction to be viewed as random.

### Procedure

The consultations at the end of medical school (T1) were carried out with a 43-year-old female simulated patient who made her first consultation with a general practitioner for irregular menstrual bleeding. The task was to carry out a complete first consultation in a general practice setting within 15 min.

The patient conveyed a medical history with multiple problems, including psychosocial distress such as recent divorce, moving to a new place, a stressful job and fear of uterine cancer, which her mother had died from 10 years earlier. This narrative was constructed through discussions within a research team of nine experienced clinicians (four general practitioners, one oncologist and four psychiatrists). Four professional actors, one from each of the four university cities, played the role of the patient. A professional acting instructor was hired to train them together to standardize the role as much as possible.

A second interview was arranged with the same students, now young physicians, about finishing the obligatory part of the postgraduate internship approximately 2 years later (T2). A clinical vignette was constructed with a correspondingly complex story and fear of colonic cancer (from which her mother was thought to have died 10 years earlier) after having observed blood on the toilet paper. Four new actors were trained in the same way as at T1.

### Instruments

#### Self-efficacy measure of confidence in using communication skills

A self-assessment questionnaire, the Oslo Inventory of Self-Reported Communication Skills (OSISCS), was developed in connection with this study. This instrument was derived from a questionnaire used to evaluate communication skills in training programs run by the Nordic Cancer Union for experienced physicians (oncologists) [17]. Typical items from that questionnaire addressed their feelings about issues such as “Breaking bad news”, “To inform the patient” and to “Disclose the patient’s important aspects”. To attune the inventory to the student and young physician population, more items were added by the authors such as (How do you feel about ...) “Showing empathy”, “Listening actively”, “Avoiding being domineering in the consultation”. In this way, we got a preliminary instrument consisting of 38 items, which was given to two classes of medical students in the middle of the curriculum in one of the four medical schools (with an integrated curriculum) (*N* = 165). To identify items with satisfactory response variability, internal consistency and internal correlation, means, reliability and Principal Component Analyses (PCA) were determined. Item-variables showed a variation in the distribution of scale-values, but all of them had satisfactory distribution curves. Cronbach’s alpha of all the 38 items was 0.93 and no further increase in alpha value would be obtained by deleting items. In the PCA, the dataset fitted to the analysis with Kaiser–Meyer–Olkin Measure of Adequacy = 0.83, Bartletts Test of Sphericity = 2477.89, d.f. = 703 and *p* < 0.001. A two-component solution was chosen based on the items loading >0.50 on one component and at least 0.40 more than the loading on the other component. In this way, 10 items loaded on the first component, forming an index labelled “Relational skills” (α = 0.87). Eight items loaded on the second component with the corresponding index labelled “Instrumental skills” (α = 0.85). The inter-correlation value between the two components was 0.24. Thus, the two-component solution from the PCA was considered to be the most salient. Accordingly, 18 items were included in the final version of the OSISCS (α = 0.85). The scores were given on a 5-point Likert type scale indicating how confident they would feel in performing 18 specific communication tasks when seeing a patient for the first time with values ranging from 1 = not at all confident (in own skills) to 5 = very confident.

#### Expert rating scales for communication skills

The Arizona Communication Interview Rating Scale (ACIR) was applied to rate communication skills from the videotapes. The ACIR consists of 14 items for scoring utterances from the physician on a scale from 1 (least) to 5 (best) [18]. Typical items were skills in: “Transitional utterances”, “Open questioning”, “Summing up”, “Eye contact”. The psychometric properties of this instrument have been found satisfactory in earlier studies [19]. The internal consistency (Cronbach’s alpha) in our sample was .86. Three trained raters scored the videotapes and a fourth rater scored one-third of them again to check the interrater reliability. The Intraclass Correlation Coefficient (ICC (1,1) between raters was found to be satisfactory (.69) [20].

After checking the correlation levels between self-assessment and observers’ ratings, we wanted, as a next step, to divide the sample according to scores on Arizona (observers’ ratings) at both T1 and T2 in three parts obtaining 1/3 low, 1/3 medium, and 1/3 high competence groups. This procedure would make it possible to investigate in more detail how the participants’ self-assessed skills would match the competence grouping. Gender was rated female = 1, male = 2, age as a continuous variable. All participants were native Norwegians and 93% of them had been assistant physicians during the last year of medical school, indicating that they should have had some extracurricular training in communication skills during this year.

### Statistics

Correlations between continuous variables were computed using Pearson’s product-moment correlations. Comparisons between groups were performed with chi-square analyses (categorical data) and one-way analysis of variance (continuous data). All statistical computations were performed using SPSS software (version 21).

## Results

Levels of self-efficacy (OSISCS) and observed communication skills (ACIR) at T1 and T2 among females, males, and in the total sample are presented in Table 1. At T1, the mean OSISCS-score was 3.50 ± .37 (scale 1–5), males had significant higher OSISCS- scores than females. For all 18 items, scores varied from 3.13 ± 1.12 (give bad news) to 4.72 ± 1.02 (present oneself). Mean ACIR-score was 3.08 ± .73 (scale 1–5), no gender difference, and on item-level scores varied from 2.31 ± 1.03 (transitional utterances) to 3.87 ± .88 (no interruptions).

**Table 1:** Self-assessment (OSISCS) and observed communication skills (ACIR) - T1 and T2 - females, males and total sample

	Female (*n* = 43) Mean SD	Male (*n* = 19) Mean SD	Total sample (*n* = 62) Mean SD	Female vs. male		
				df	t	p
T1						
OSISCS Total	3.42 + 0.31	3.67 + 0.45	3.50 + 0.37	59	2.53*	
ACIR	3.12 + 0.70	3.01 + 0.80	3.08 + −.72	60	.53n.s.	
Pearson’s r OSISCS/ACIR	.032, n.s.	−.186 n.s.	−.082 n.s.			
T2^a^						
OSISCS Total	3.55 + 0.46 n.s.	3.70 + 0.40 n.s.	3.60 + 0.49 n.s.	60	.64 n.s.	
ACIR	3.67 + 0.67 ***	3.27 + 0.60	3.55 + 0.67 ***	60 2.26*	60 2.26*	
Pearson’s r OSISCS/ACIR	−.327*	−.291 n.s.	−. 34 **			

*n.s.* not significant, *p < .05, **p < .01, ***p < .001

^a^Degree of statistical significance with t-test scores from T1 and T2 in both gender groups separately and in the total sample (vertical) is indicated after the value at T2

At T2, mean OSISCS-score was 3.60 ± .49, no gender difference, single items varying from 2.95 ± .82 (help patient master their disease) to 4.35 ± .89 (take an admission record). Mean ACIR-score was 3.55 ± .67, females significantly higher than males, single items varying from 2.56 ± 1.15 (additional questioning) to 4.32 ± .95 (no interruptions).

At T1, no significant correlation was found between self-assessment scores (OSISCS) and the observers’ ACIR scores. At T2, however, there was a significant negative correlation between the mean observed ACIR score and mean total OSISCS score (*r* = − .34, *p* < .01), female physicians contributing to this significant negative correlation.

**Fig. 1:**
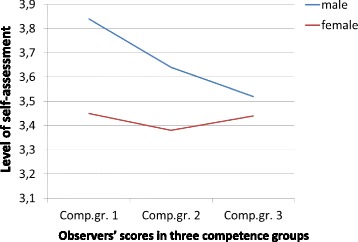
Level of self-assessed self-efficacy in males/females across competence groups at T1. Legend (horizontal) - Observers’ scores in three competence groups (vertical) - Level of self-assessment

In order to investigate how the negative correlation between self-assessed self-efficacy and observed communication skills at T2 could be explained, we divided the sample into three groups based on the observers’ ratings (low, medium, and high communication competence) Fig. 1 shows the OSISCS scores in the three groups for males and females with a significant difference between them across competence groups (F = 6.39, *p* = .014), males with highest level of self-efficacy self-assessment.

**Fig. 2:**
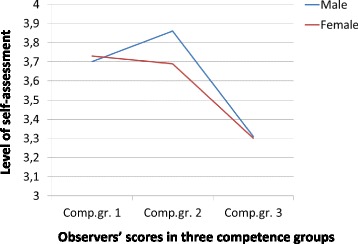
Self-assessment in female and male physician according to performance group – T2. Legend (horizontal) - Observers’ scores in three competence groups (vertical) - Level of self-assessment

At T2 (Fig. 2), a different pattern occurred with high self-efficacy assessment scores for both genders in the low- competence group, close to the score of the middle- competence group, but in the high competence group, the self-assessment scores were significantly lower in the high competence group compared with the two other groups (F = 6.35, *p* = .003) with similar levels for both genders.

## Discussion

In this study, we have investigated the relationship between self-assessed self-efficacy and observers’ ratings of competence in clinical communication at T1 and T2. At T1, male medical students considered their self-efficacy in communication tasks to be significantly higher than female students, while there were no differences between the genders in the level of observers’ ratings of actual communication skills. At T2 however, females were rated by the observers as more competent in communication skills than males, while the mean level of self-assessment did not differ. Moreover, while the two measures were uncorrelated at T1, a significant inverse relationship was found at T2. This difference over the period of internship was somewhat surprising as we had expected that with increasing clinical experience, young physicians would demonstrate more mature and realistic self-assessment which potentially could imply a positive correlation between the two measures. When this assumption did not hold true, we have to answer the question posed in the title with – no -, medical students and young physicians do not assess their self-efficacy in communication skills realistically and reliably.

The general tendency among women to under-estimate and among men to over-estimate their communication skills have been found in some studies [21, 22] and was replicated at T1 in our study. At T2, however, this difference between the genders disappeared and another pattern appeared. Both female and male physicians disclosed the same relationship between the two assessment methods at T2 across competence levels. But the negative association between the two measures was restricted to the sub-group with the highest communication skills as rated by expert observers.

It can be reasonable to interpret this finding as an increase in self-criticism concerning communication skills among the physicians of both genders. Those who displayed and developed high competence in communication skills throughout internship may have attained a better understanding about communication with patients being a demanding task. Thus, they could turn out to be somewhat more critical in judging their own self-efficacy in communicating with patients after the period in which they met “hard” reality after “sheltered” medical school position.

As indicated in the Background section, there are conflicting results presented in the literature on the concordance between self-assessed and observed skills. An inverse relationship between these two measures has been observed previously [3], but not in a study with a design like ours with the possibility to investigate the course over a period of one-and- half to 2 years. The combined results of this design have shed light on both competence and gender aspects over the period.

These results go beyond what is presented in the literature including an earlier study within our research group showing that during the internship period, young female physicians significantly improved their communication skills, as assessed by independent observers, while male physicians did not [23].

The important question raised from our results is whether the level of self-criticism we have found within the high performance group after internship is productive or contra-productive for further learning. As an assessment of communication skills, the self-reported self-efficacy is, as mentioned above, not accurate, neither at T1 nor at T2. However, viewed in the perspective of a personal development it can be a step in the course of more realistic awareness of obtained skills. But for the low and medium competence groups this maturing process seems not to be present over the relatively short time span this study has covered.

Thus, a challenge for curriculum and post-graduate training planners will be to arrange training facilities in which medical students and young physicians will get the opportunity to confront their self-perception with observers’ ratings of their competence in communication with patients.

A future study within the same cohort with a longer follow-up period (for instance 10 years after graduation) could perhaps reveal somewhat more concordance between the two assessment methods. Longer time with more knowledge, experience and skills may lead to a more secure identification with the role of doctor [24] reducing the gap between a self-critical attitude and real performance.

### Strengths and limitations

The strength of this study is the prospective design with repeated measures before and after internship of both self-efficacy assessments and expert ratings of communication skills, giving us the opportunity to study the correspondence between the two methods across gender and time-points.

There are also some limitations. The sample is small, and the representativeness of medical students and young physicians at large is of course uncertain, even though there was satisfactory correspondence in scores both on self-efficacy and observed skill between the larger sample of the 111 students attending at T1 and the 62 young doctors among the 111 yielding data in the second procedure they attended [23]. The 111 participants out of 320 eligible students in the first procedure might have been among those being most motivated for training and testing their level of obtained communication skills. If this is the case, our results can be biased from a Type I error. The reduction from 78 to 75 is minor, and the reduction from 75 to 62 we view as random as it was due to logistic and ordinary mail delivery problems. The single case history and consultation design is also a limitation which can reduce the generalizability. In addition, the raters were not blind for T1 or T2, which also can have biased the results in a Type I error direction.

Our data being around 10 years old may yield another picture of students’ confidence in their own skills than would have been found today. A recent study we have performed by comparing data from 2004 and 2015 upon students attitudes towards communication skills in two of the four Norwegian universities show very similar results over this 11 years’ time span (Gude - unpublished data).

Moreover, the comparison between general self-efficacy scores and scores from observers concerning clinical communication skills may be questionable due to our instruments covering different aspects. The questionnaire (OSISCS) specifically asks for scoring of self-efficacy, which may be viewed as something different from perceived competence in these skills during a specific consultation. All the way, we view our findings as highly indicative answers of the questions we have raised.

## Conclusions and implications

The lack of positive correlations between self-efficacy assessment and expert ratings points to limitations in the applicability of self-assessment measures of communication skills. Due to gender differences, groups of female and male physicians should be investigated separately. Those obtaining high-performance ratings from observers, through the period of internship, may become more conscious of how demanding clinical communication with patients may be. This insight may represent a potential for growth, but could in some physicians represent too much of a self-critical attitude. Active supervision of young physicians throughout internship is important in order to help them to be more aware of their strengths and weaknesses, in order to gain increased mastery in the art of doctoring.
